# Recent Advances of Plasmonic Organic Solar Cells: Photophysical Investigations

**DOI:** 10.3390/polym10020123

**Published:** 2018-01-26

**Authors:** Lin Feng, Mengsi Niu, Zhenchuan Wen, Xiaotao Hao

**Affiliations:** 1 School of Physics, State Key Laboratory of Crystal Materials, Shandong University, Jinan 250100, Shandong, China; lfeng@sdu.edu.cn (L.F.); niumengsi@163.com (M.N.); xiaoheshangsdu@126.com (Z.W.); 2 ARC Center of Excellence in Exciton Sciences, School of Chemistry, the University of Melbourne, Parkville, Victoria 3010, Australia

**Keywords:** photophysics, organic photovoltaics, plasmonics

## Abstract

The surface plasmon resonance (SPR) of metallic nanomaterials, such as gold (Au) and silver (Ag), has been extensively exploited to improve the optical absorption, the charge carrier transport, and the ultimate device performances in organic photovoltaic cells (OPV). With the incorporation of diverse metallic nanostructures in active layers, buffer layers, electrodes, or between adjacent layers of OPVs, multiple plasmonic mechanisms may occur and need to be distinguished to better understand plasmonic enhancement. Steady-state photophysics is a powerful tool for unraveling the plasmonic nature and revealing plasmonic mechanisms such as the localized surface plasmon resonance (LSPR), the propagating plasmon-polariton (SPP), and the plasmon-gap mode. Furthermore, the charge transfer dynamics in the organic semiconductor materials can be elucidated from the transient photophysical investigations. In this review article, the basics of the plasmonic mechanisms and the related metallic nanostructures are briefly introduced. We then outline the recent advances of the plasmonic applications in OPVs emphasizing the linkage between the photophysical properties, the nanometallic geometries, and the photovoltaic performance of the OPV devices.

## 1. Introduction

In the photovoltaic industry nowadays, the inorganic solar cells based on poly- and mono-crystalline silicon account for more than half of the market share. The efficiencies of the silicon solar cells are typically 15–18%, and the thickness is in the range of 160–240 μm [1]. However, the inorganic solar cells are expensive as a result of the high-cost and complicated processing of Si. Therefore, new generations of solar cells are in great demand. In recent years, organic photovoltaic cells (OPVs) have garnered much research interest, because they are promising candidates for a next-generation renewable power source due to their advantages such as being low-cost, light-weight, and having mechanical flexibility. Up to date, the power conversion efficiency (PCE) of OPV has been as high as 13–14% [2,3,4], demonstrating its potential for final commercial productions.

In OPVs, owing to their relatively low carrier mobility (on the order of 10^−4^ cm^2^/V·s) and short diffusion length in most of the organic semiconductor materials [5], the photoactive layer is usually required to be quite thin, i.e., around 100 nm or less, to facilitate the charge carrier diffusion and extraction [6]. It results in poor absorption of the incident light, while much photon energy that could have been converted to the electrical energy is lost. Hence, intensive research has been stimulated to further enhance the light absorption of OPVs.

To date, many studies presented that without varying the thickness of the photoactive layer, the surface plasmon resonance (SPR) effect can be introduced via the involvement of metal nanostructures to boost the light harvesting in the photoactive layer, thereby enhancing the photovoltaic performances [7,8]. The shapes, sizes, and compositions of the metallic nanoparticles (NPs) can be chemically tailored to manipulate the plasmonic enhancement of the optical absorption [9,10,11].

Between the structural configurations of the plasmonic NPs and the ultimate device performance, there is an important intermediate link, which is the photophysics of the system [12,13,14]. By monitoring the photophysical properties by means of steady-state and transient absorption or photoluminescence spectroscopy, the charge transfer dynamics and detailed plasmonic processes can be investigated.

This article gives an overview of the advances in plasmonic-enhanced OPV solar cells in recent years and emphasizes the involved photophysics. Firstly, the mechanisms of the surface plasmon resonance (SPR) effect are introduced. Then, the configuration and location of the diverse plasmonic nanostructures are summarized in association with relevant device performance enhancement. We show that the steady-state and transient photophysical characterizations have been widely used to clarify the plasmonic processes and operating principles in boosting the OPV performances. Finally, conclusion and outlooks based on plasmonic OPV are listed out.

## 2. Mechanism of Plasmonic Enhancement Effect in Opvs

**Figure 1:**
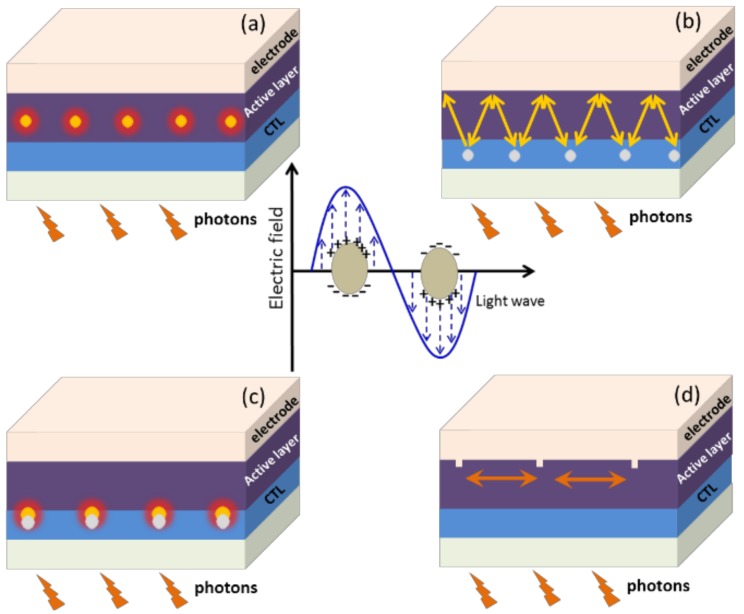
Architecture of OPV solar cells with plasmonic metallic NPs. (**a**) NPs in the active layer for LSPR enhancement; (**b**) NPs in the charge carrier transport layer (CTL) as light trapping centers due to the forward scattering effect; (**c**) NPs in CTL that induced the enhancement of the electromagnetic field in the photoactive layer due to the LSPR effect; (**d**) light trapping by the excitation of surface plasmon polaritons (SPP) at the metal/semiconductor interface. The center graph is a mechanism illustration of localized surface plasmon in metallic NPs.

Adequate light absorption is prerequisite to the OPV devices for the improvement of PCE. The light trapping in the organic solar cells can be intensified by incorporating metallic NPs. At the metallic-dielectric interfaces in multiple plasmonic solar cell configurations, the electrons of the nanometals can be excited collectively, which amplifies the electromagnetic field in the photoactive layer of the device (Figure 1). This is called SPR effect and can be elucidated by the Mie theory [15,16].

The plasmonic enhancement can be achieved through several different SPR mechanisms, e.g., the far-field scattering effect (Section 2.1), the near-field localized or propagating SPR modes (Section 2.2), and some other plasmonic modes like waveguide mode and plasmon-cavity mode [17,18,19,20,21] (see Figure 1). The enhancement of optical absorption can be achieved by far-field scattering effect due to the increase of the optical path of incident photons and the decrease of the reflection at illuminated surfaces. In cases of near-field SPR effect, the number of absorption events is increased, because the metallic nanostructures can confine electromagnetic waves at the metal-dielectric interface and produce intense near field.

### 2.1. Far-Field Scattering Effect

Upon the far-field scattering, the optical path of the incident sunlight is lengthened, which may promote the absorption of the photons. The size of the metallic NPs that best fits the forward scattering effect usually falls in the regime of 30–60 nm [22].

For plasmonic nanostructures embedded in front buffer layer (i.e., HTL in conventional type or ETL in inverted type solar cells), the scattering in the forward direction is requisite. The geometry of the plasmonic nanostructures is optimized to minimize the reflection at the surfaces. The optical paths of the incident sunlight in the absorbing medium and their interaction time are thus increased, resulting in enhanced absorption efficiency of the photoactive layer [23]. For the NPs in back buffer layers further away from the incident photons, backward scattering should be manipulated to the maximum for efficient light absorption.

The scattering direction can be tuned by controlling the size and geometry of the plasmonic nanostructures. From NPs with a diameter of c.a. 50 nm, photons are scattered forward and backward in comparable quantities [24]. As the particle size increases, more radiation is scattered in the backward orientation.

NPs with sizes smaller than abovementioned intermediate range present weak scattering, which induces insufficient optical absorption. Moreover, the conductivity of the device is detrimentally affected because more charge trapping sites are formed at the NP sites due to their large surface-to-volume ratio. For plasmonic nanostructures embedded outside the active layer, such losses overwhelm the possible advantages that small NPs (e.g., ~20 nm) may exaggerate the coupling of the near plasmonic field to the active layer [25]. Likewise, overly large nanostructures (e.g., hundreds of nanometers) are also harmful in view of the inferior layer morphology, strong absorption inside the metal, and shunting problems [26].

### 2.2. Near-Field Localized and Propagating Plasmonic Effects

#### 2.2.1. Localized Surface Plasmon Resonance (Lspr)

The localized surface plasmon resonance (LSPR) is non-propagating excitation of electrons confined in a metallic NP. When LSPR occurs, the electrons inside metallic NPs collectively oscillate with the incident radiation [27], leading to greatly intensified electric fields near the NPs surfaces. LSPR often takes place when the surfaces of the plasmonic nanometals are curved or kinked [28].

The optical absorption reaches its maximum at the plasmon resonance frequency. The resonance frequency is geometry-dependent for the metal nanostructures. Therefore, tailoring appropriately the size and morphology of the NPs is an effective approach to control the plasmonic enhancement to meet specific demands. For noble metal NPs, the resonance usually occurs in the visible and near infrared wavelength region [29,30].

**Figure 2:**
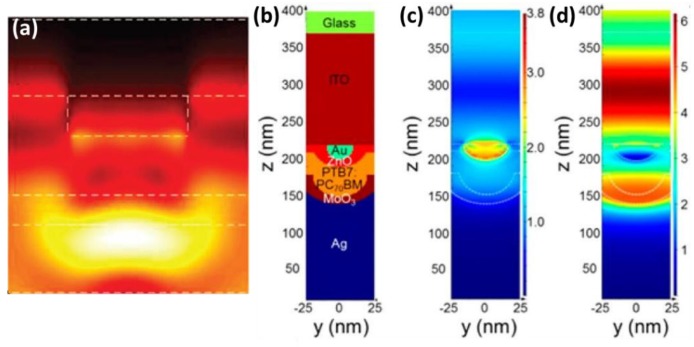
(**a**) Waveguide model at the wavelength of 400 nm [35]; (**b**) device material profile; and (**c**) electric and (**d**) magnetic field intensity profiles on Au NPs at 560 nm [37]. Reprinted with permission from Ref. [35] and Ref. [37].

Normally, the size of the metal NPs should be smaller than the wavelength of the incident light for plasmon excitation. The NPs with sizes smaller than 30 nm act as subwavelength antennas upon the LSPR excitation. In such cases, the absorption enhancement is dominantly from the LSPR effect rather than the scattering effect [31]. Metallic NPs, which are smaller than 30 nm, behave as local field enhancers instead of scattering centers. The plasmonic near-field induced in the photoactive layer increases the absorption cross-section and thus effectively enhances the photon absorption. If these kinds of NPs are placed outside the active layer, e.g., in the buffer layer (see Figure 2), the enhanced electric field may also be coupled to the absorbing layer, increasing its effective absorption cross section [32,33].

#### 2.2.2. Propagating Surface Plasmon Polariton (Spp)

In contrast to LSPR, surface plasmon polariton (SPP) often occurs on or in vicinity of planar metal films. SPP is an electromagnetic wave at infrared or visible frequencies that propagates in a wave-like fashion along the planar metal-dielectric interface [34,35,36]. The charge motions of SPP involve contributions from both the metal and the dielectric. The wavelengths of SPP waves are shorter in comparison with the incident irradiation, resulting in more intense magnetic field. The amplitude of the SPP magnetic waves decays exponentially with increasing distance into each medium from the interface. Perpendicular to the interface, the spatial confinement of SPP is in a subwavelength-scale range. An SPP can propagate along the interface for 10–100 μm until its energy is consumed by the absorption or scattering. This increased optical path with the lateral propagation promotes the absorption significantly.

### 2.3. Some Other Modes for Absorption Enhancement

#### 2.3.1. Photonic Waveguide Mode

Besides SPP mode, another kind of waveguide mode may be excited at the planar metal/dielectric interfaces upon the incident irradiation, which also contributes to the absorption enhancement. It is called “waveguide mode” because it resembles the propagation pattern in slab dielectric waveguides (Figure 2). Along its lateral propagation, part of the waveguide mode can be absorbed by the organic semiconductors, in which excitons are generated [38,39]. The simulation of the waveguide mode can be achieved by solving Maxwell’s equations governing optical properties of solar cells by using the finite-difference frequency-domain (FDFD) method.

The excitation of the waveguide mode is independent on the polarization of the incident irradiation. In another word, there can be waveguide modes regardless of whether the polarization direction of the excitation is parallel with or orthogonal to the metal nanopattern directions [40].

#### 2.3.2. Plasmon-Cavity Mode

When specific metal-dielectric configurations are constructed, the spectral wavelength ranges for absorption enhancement can be further expanded due to the plasmonic coupling modes. For instance, a metal-dielectric-metal (MDM) structure is obtained when the photoactive layer is sandwiched by the metal electrode and metallic NPs layer. By appropriately selecting the NPs geometries, standing waves of the surface plasma can be generated as a result of constructive interferences in the finite cavity, which decreases the light leakage from the surface plasmon generation in lateral gaps between the NPs. Thus, the plasmon-cavity mode is excited in MDM and strong electromagnetic field is confined into the light absorbing layer. It is worth noting that the field enhancement from the plasmon-cavity plasmonics is independent of the polarization or incidence angles of the incoming light [41].

## 3. Material Nanostructures for Plasmonic Enhancement

Up to date, the metals most often adopted for plasmonic enhancement in bulk heterojunction (BHJ) OPV solar cells are gold and silver, because the plasmonic resonance is readily observed in Au and Ag due to their delocalized valence electrons [42]. However, in consideration of the high-cost of Au and Ag, it is also important to use inexpensive plasmonic materials in organic solar cells, since a major advantage of organic photoelectric conversion system is the low cost. The utilization of copper and aluminum for plasmonics have also been reported in the literature [43].

**Figure 3:**
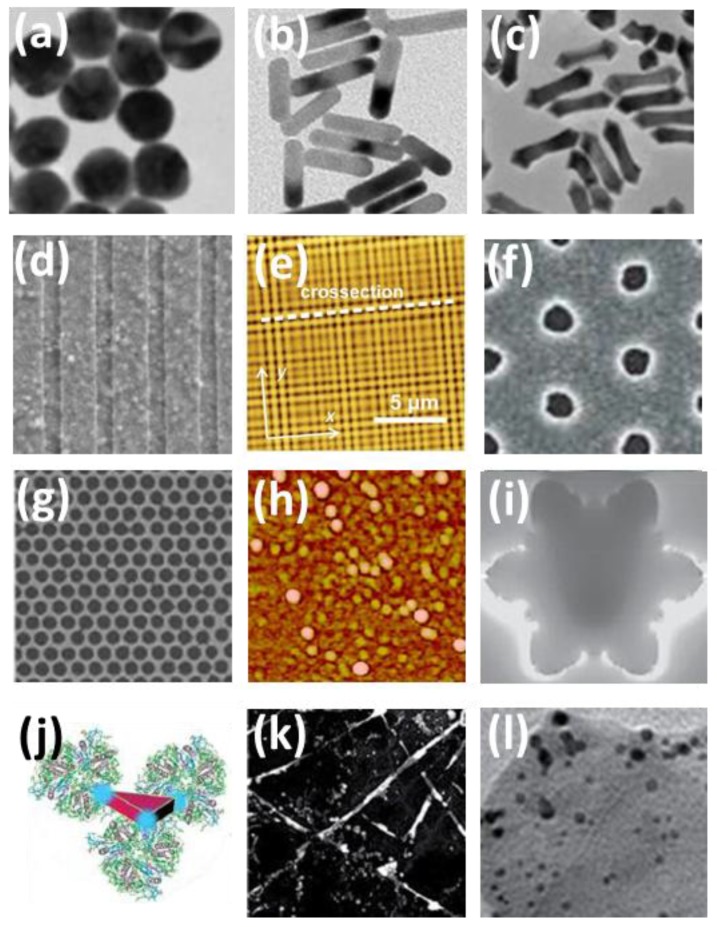
Some metallic nanostructures for plasmonic enhancement, including (**a**) NPs, (**b**) Nanorods (NRs), (**c**) nanoarrows, (**d**) grating, (**e**) multidiffractive grating, (**f**) nanohole array, (**g**) nanomeshes, (**h**) bimetallic NPs, (**i**) asymmetric nanostars, (**j**) nano-bio hybrid, (**k**) Ag nanowire networks, and (**l**) NPs linked with 2D material WS_2_. Reprinted with permission from Refs. [51,52,53,54,55,56,57,58,59,60].

The spectral position of the LSPR peak can be controlled by tailoring the shape, size, and spacing of the nanometals and the dielectric environment. The shapes of the particles can be fabricated to be spheres, ellipsoids, hemispheres, prisms, cylinders, and anisotropic structures. When metals are used to construct the electrode, they can be patterned to gratings, nanohole arrays, and nanomeshes (Figure 3) [41,42,44,45]. The LSPR peaks of the metallic NPs arise from multiple orders of dipole modes and usually have narrow bandwidths for plasmonic absorption enhancement. In spherical NPs with identical sizes, Au NPs whose permittivity is larger exhibit LSPR peaks at longer wavelengths with respect to Ag NPs [46].

In order to expand the spectral region of the plasmonic absorption enhancement to wide visible or even to infrared regime, one needs to incorporate multiple metals or metals combined with organic molecules into the OPV devices [47,48,49]. For instance, broadband SPR enhancement can be achieved by mixing Au and Ag NPs in one layer or stacking multiple metallic NPs layers. The nano-bio hybrids with Ag nanoprisms and natural sensitizer molecules allow the harvest of photons in a broad spectral band due to the plasmonic light trapping from Ag nanoprisms and the energy transfer from the natural solar energy absorbing protein.

Another alternative approach is the employment of nanometals with complex and asymmetric geometries [9,50]. In such anisotropic nanostructures, these exist in an inherent coupling between the center parts and the surrounding branches, which generates concentrated field with high intensity and remarkable scatterings. This novel methodology enables broadband plasmonic resonances and has great potential to enhance the photovoltaic performance of OPV devices.

Metallic NPs in the active layer may serve as combination centers for the electrons and holes and dampen the OPV performances. A solution is to encapsulate the nanometals with non-absorbing shells like TiO_2_ or SiO_2_. Theoretical simulations have shown that even very thin layers may attenuate the plasmonic intensity of the metals [61]. Hence, there is a tradeoff between the losses in the electrical current and the plasmonic effect attenuation.

In recent years, two-dimensional (2D) atom-thick materials have attracted attention due to their fantastic thermal, optical, and electric properties [62,63,64]. 2D materials have been adopted in OPV devices to work concomitantly with metallic nanostructures to advance the stability and solar energy harvest. Lee et al. transferred graphene both on top and at the bottom of Ag NRs networks to protect them against thermal degradation. Constructive interference of the incident light in grapheme-Ag-graphene produced better transmittance than pure Ag NRs.

It is of great importance to point out the plasmonic effect in 2D materials. The SPR frequency of grapheme resides in the range of 10^12^~10^14^ Hz. It has been reported that grapheme plasmons can be excited at visible wavelength [65]. However, even they were involved in the solar cells, the plasmonics from the 2D materials have scarcely been discussed. Viable approaches still need to be developed to unambiguously discriminate the plasmonics of non-metal materials from those of the metals.

## 4. Device Architectures for Plasmonic Enhancement

The metallic NPs in OPV devices play different roles depending on their spatial arrangement across the device cross-section [66,67]. Nominally, the nanometals distribute in the active layer, and/or in the charge carrier transport layer, in the electrodes, or between the above mentioned layers. The configuration of the nanostructures, the relevant plasmonic mechanisms, and the performance of the corresponding organic solar cells are summarized in Table 1.

### 4.1. In the Active Layer

In BHJ solar cells, plasmonic NRs/NPs can be blended with the organic semiconductor donor and acceptor in the active layers. When the sizes of the nanostructures are smaller than ~30 nm, the LSPR effect plays the main role in achieving better absorbance. Concurrently, the incident irradiation may also be scattered by the NRs/NPs to increase the propagation length that leads to the absorption enhancement.

Theoretical calculations demonstrate that, as the NPs increase from 10 to 80 nm in size, the corresponding LSPR wavelength changes from 413 to 446 nm for Ag NPs embedded in P3HT:PCBM. For Au NPs in the same environment, the resonant wavelength falls in the range of 626–641 nm [82].

From the comparison of the plasmonic effect of silver and gold NRs/NPs with average sizes of 50 nm [51], one can see that the optical absorption and the charge carrier transport can be improved by all of the Au/Ag NPs/NRs embedded in the active layer. Finite difference time domain (FDTD) calculations based on Mie scattering suggest that the enhancement arises from LSPR and the efficient light scattering from the Ag and Au nanostructures. The materials and the nanostructures indeed influenced the exact extent of the performance boost. Au NRs/NPs outperformed their counterpart geometries of silver. For a fixed type of metal, NRs are superior to NPs, because the LSPR spectrum covers a broader wavelength range for NRs due to their synergistic transverse and longitudinal resonances [83].

**Figure 4:**
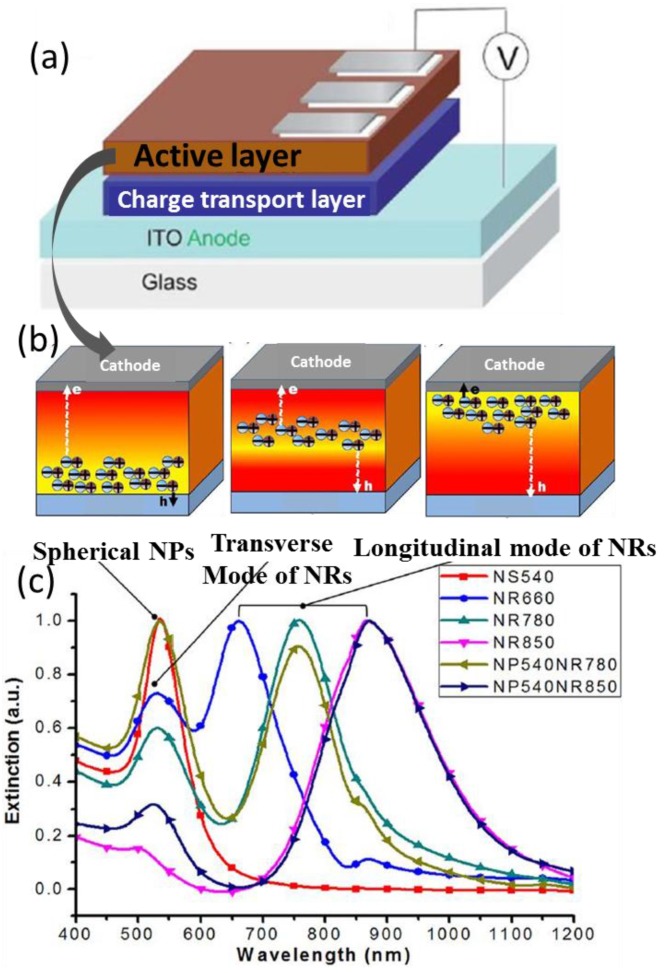
(**a**) Schematic illustration of the sandwich-type BHJ solar cell; (**b**) Ag NPs located in sublayers of the active layer [84]; (**c**) Up: Dipolar oscillations in spherical Au NPs and Au NRs. Bottom: normalized UV-Vis-NIR absorption spectra of Au NPs of various sizes and shapes in water [83]. Reprinted with permission from Refs. [83,84].

When the thickness of the active layer is large enough, the NP ensemble possibly distributes in certain sub-layers (Figure 4). It may locate near the cathode/anode, or in the middle. In such cases, the location of photoinduced excitons and thereby the transport path of charge carriers may be optimized in consideration of electrical properties of the charge carriers [66]. Sha et al. investigated the impact of the sub-layer dispersion on the photovoltaic performance of small-molecule organic solar cells. Since the hole mobility of CuPc:C_60_ is about four orders of magnitude smaller compared to the electron mobility, the optimal charge transport path was obtained with Ag NPs in the active layer near the anode. While free electrons and holes are generated in the exciton region near the anode, the transport path of the low mobility hole to the anode is shortened [84].

Despite the fact that Au NRs serve as a good selection for plasmonic enhancement, some studies pointed out that bare metal NPs/NRs surfaces may act as recombination centers for holes and electrons, which may deteriorate their photovoltaic characteristics [85]. A proposed solution is to cap the metal NPs/NRs with inorganic materials to form core-shell structures [86]. Yamada et al. demonstrated that Ag NPs with diameters of 46–60 nm that were encapsulated with a 2 nm thick TiO_2_ shell resulted in the shift of plasmonic resonant peak from 430 to 438 nm, because of the relatively high refractive index of TiO_2_ (>2.4). The external quantum efficiency (EQE, which equals to photon conversion efficiency (IPCE)) of the solar cells presented the maximum enhancement at wavelengths near the LSPR peak of Ag NPs@TiO_2_.

Apart from being placed in the active layer alone, a novel strategy is to first link the metallic NPs onto 2D materials before the incorporation. 2D laminar materials have drawn considerable attention due to their extraordinary properties and great potential for various applications [87].

When the Ag NPs are in the photoactive layer, the PCE enhancement is usually correlated with the *J*_sc_ increment, which principally arises from the near-field LSPR effect. While the Ag NPs are placed in the electron transport layer, the increase of PCE may come from two aspects. One is analogously the *J*_sc_ increment due to the plasmonic effect. The other is the increment of fill factor (FF) and open-circuit (*V*_oc_), which is caused by the electrical resistance reduction due to the Ag NPs.

**Figure 5:**
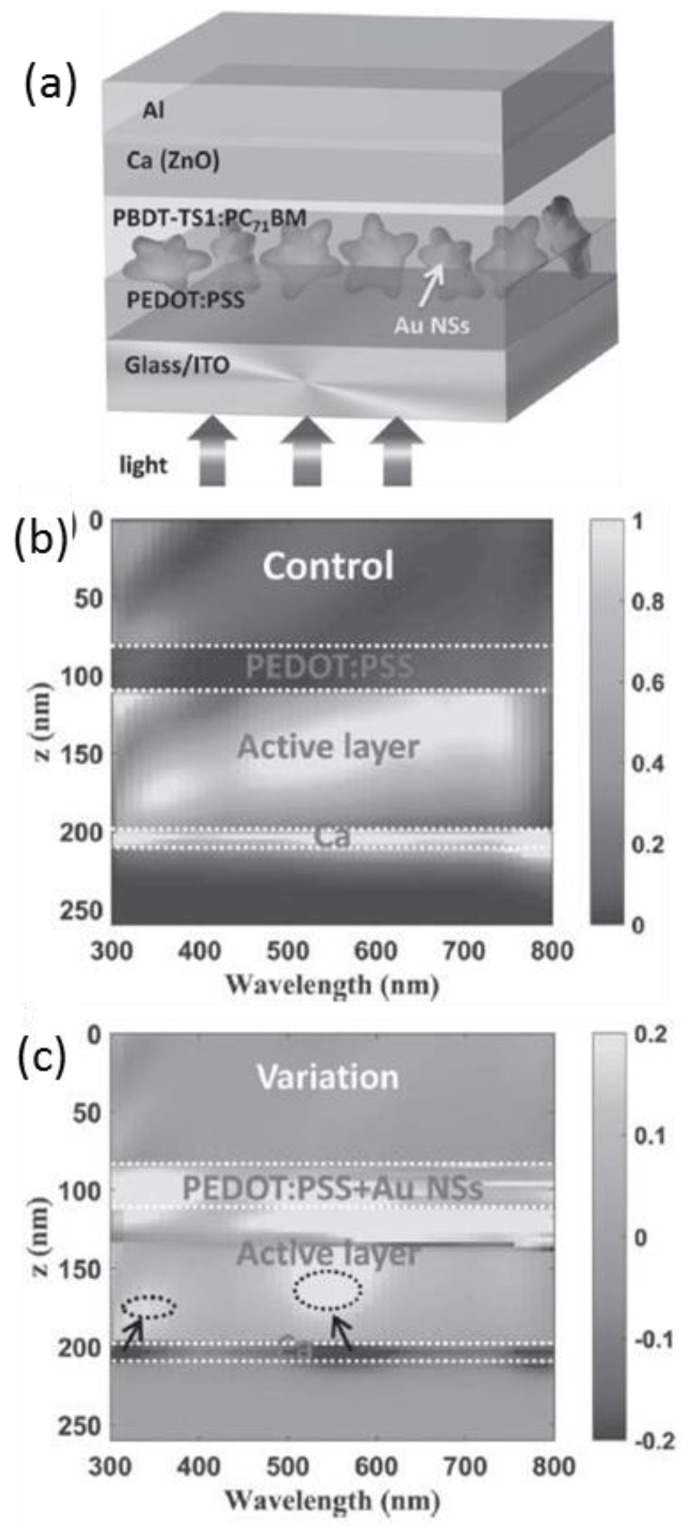
(**a**) The schematic illustration of the structure of Au nanostars (NSs) incorporated OSC; (**b**) the absorptive power distributions of the control OSC (without Au NSs). The absorptive power in Ca cannot contribute to the carrier generation and would be wasted; (**c**) the variation of absorptive power distribution due to the incorporation of Au NSs compared to the control OSC [53]. Reprinted with permission from Ref. [53].

The utilization of asymmetric metallic nanostructures, e.g., nanostars, is a simple and promising approach to achieve a broadband absorption enhancement (Figure 5). The plasmonic asymmetric modes that arise from the core-branch coupling significantly increase the local field intensity, thereby enhancing the absorption in the active layer. The Au nanostars also help transfer the optical power in the charge transport layer (which otherwise will be a waste) to the light absorbing medium and thus improve the absorption. The absorption peaks from the high-order asymmetric plasmonic modes are spectrally consistent with the EQE enhancement, pushing the PCE up to 10.5% [53].

### 4.2. In Charge Transport Layer

Despite the fact that positive effects have been reported for the OPV devices with the plasmonic nanostructured embedded in the photoactive layer, one should note that some negative side effects may be introduced by them into the photovoltaic performances. Firstly, the surfaces of nanometals may function as the recombination centers for charge carriers, decreasing the number of free charges towards the collection at the electrode [73]. Secondly, the nanostructures do not form continuous network and the charge transport between the nanometals and the acceptor semiconductor could be worse than in the continuous network of the electron acceptor material [88].

**Figure 6:**
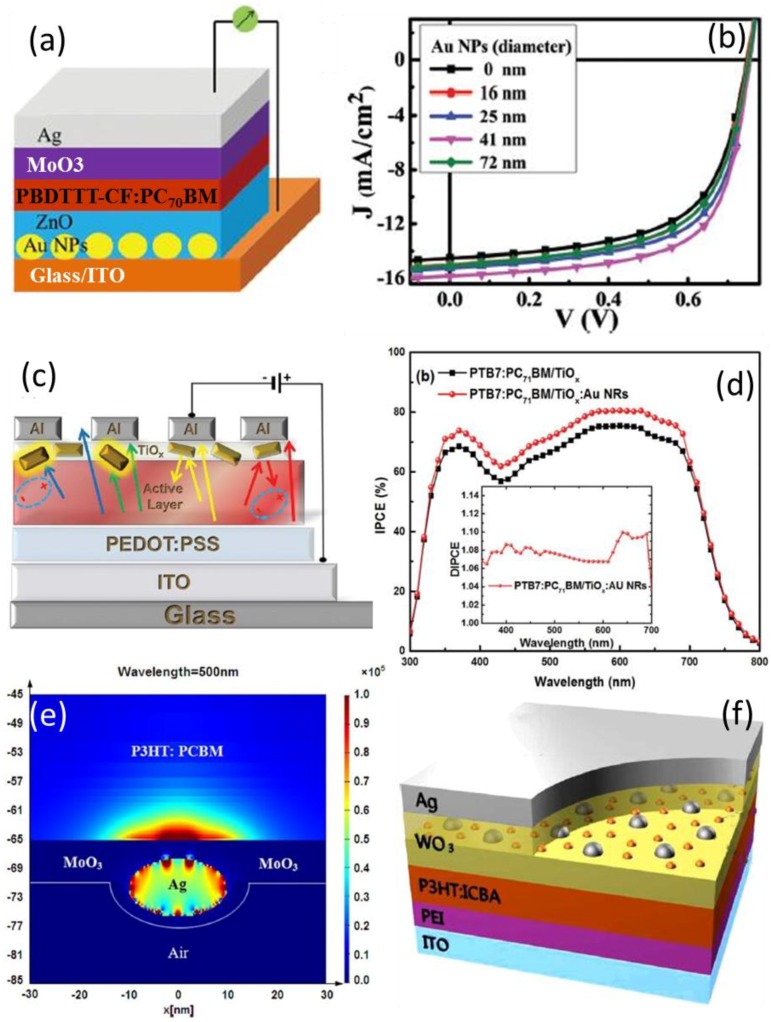
Forward (**a**,**b**) and backward (**c**,**d**) scattering when Au NPs/NRs are embedded in ETL [70,73]; (**e**) LSPR mode, which is generated in vicinity of metallic NPs in CTL and scattered into the active layer [89]. (**f**) Bimetallic NPs embedded in HTL [54]. Reprinted with permission from Refs. [54,70,73,89].

To avoid those disadvantages, the plasmonic nanostructures have been placed in the charge transport layers (CTL), namely, the electron transport layer (ETL, i.e., cathode buffer layer) or hole transport layer (HTL, i.e., anode buffer layer). The scattering may be forward or backward depending the specific location of the nanostructures. When the nanometals are embedded in the front CTL (Figure 6a), the forward scattering effect is mainly utilized to increase the optical path length and the absorption in the active layer. For nanostructures located in the rear CTL (Figure 6b), the incident irradiation is back scattered by them into the active layer to lengthen the optical path. NPs/NRs located in the rear CTL of OPV devices may suppress the energy losses by the absorption of the metals and by the back-scattering of light, which would be unavoidable when they are in front CTL [70].

Au NRs/NPs with sizes around 40 nm doped in ETL like TiOx or ZnO can significantly enhance PCE by ~20% to about 7.86% relative to the control device [70]. The PCE enhancement is attributed to light trapping mainly caused by the plasmonic far-field scattering. Furthermore, simulations revealed that the electromagnetic field can be enhanced in the active layer even when the nanometals totally locate in CTL [89].

In addition to the conventional NPs/NRs, other types of nanostructures have been explored, such as nanoarrows, or NPs hybridized with carbon nanotubes (CNT). Incorporating Au nanoarrows into the ZnO ETL can result in lower resistance and improved *J*_sc_ and EQE. The optimized doping concentration of 1.5 wt % led to a 27.3% increase of PCE [72]. ZnO@CNT-Au, the hybrid of ZnO NPs and Au NP-decorated carboxylic CNTs, has been prepared as ETL. Au NPs induce the surface plasmon effect to increase light absorption. CNT provides a template for the in situ growth of ZnO NPs, forming a homogeneous film with fewer defects and higher conductivity. By employing ZnO@CNT-Au as cathode buffer layer, the charge recombination was decreased and electron collection was increased. Consequently, the device with ZnO@CNT-Au showed a significant enhancement of PCE up to 7.9%, which provides a promising method to improve the performance of PSCs [59,71].

On the other hand, metallic NPs have been doped in HTL such as PEDOT:PSS, WO_3_, or MoO_3_ [54]. For instance, dual Au-Ag NPs in the WO_3_ HTL extended the wavelength range of light absorption due to the backward scattering and LSPR effect.

**Figure 7:**
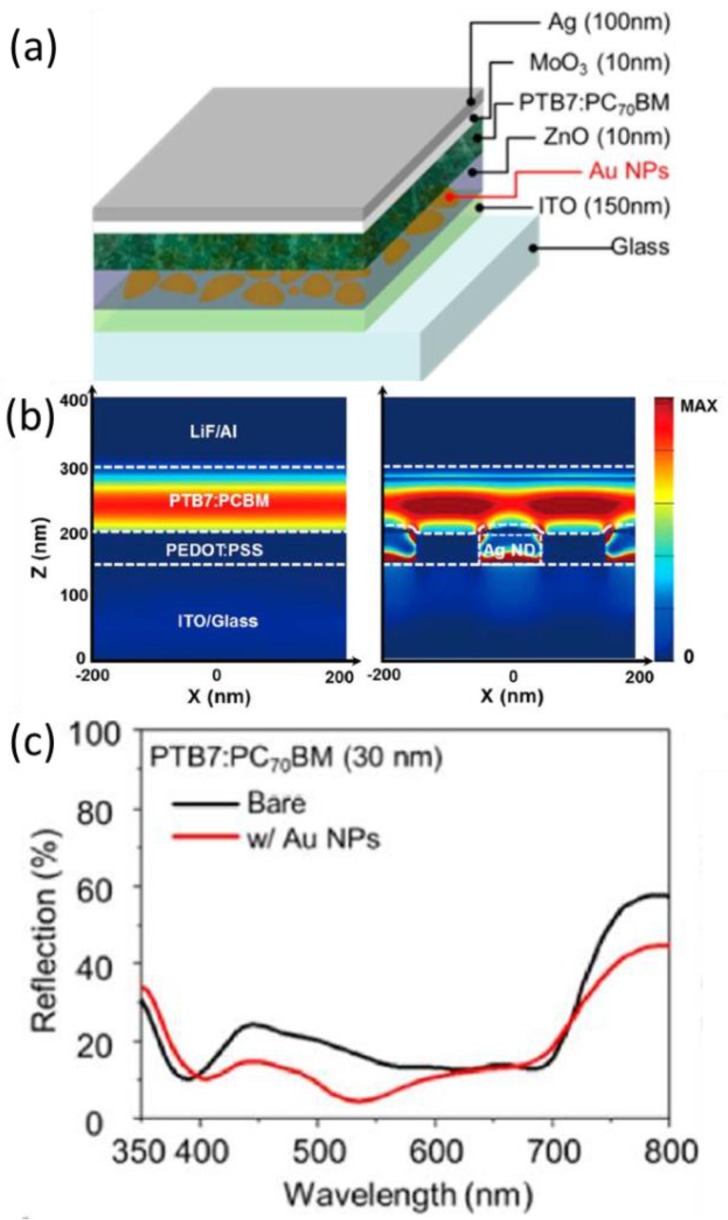
Plasmonic effect of MDM structures. (**a**) Schematic of organic photovoltaic device structure with Au NPs in front ETL; (**b**) absorption density profiles in OPV devices with and without Ag NDs at the resonance wavelength (λ = 573 nm) [76]; (**c**) reflection spectra from PTB7:PC_71_BM layer [37]. Reprinted with permission from Refs. [37,76].

When the nanometals are placed in the front CTL, i.e., HTL of the conventional type or ETL of the inverted type solar cells, and when the back is the metal electrode, the MDM configurations are constructed in which the electric field can be strongly confined in the middle dielectric materials (Figure 7). Such a structure is beneficial for the efficient absorption of sunlight in the photoactive layer with possible plasmonic mechanisms involved such as forward scattering, LSPR, plasmon-cavity mode, and plasmon-gap mode [75,77]. The nanometals are dispersed comparatively randomly in CTL if they are first blended in the precursor solution for CTL before the spin-coating. Alternatively, periodic metallic nanostructures can be first fabricated on ITO before the formation of CTL [55].

### 4.3. Between the Active Layer and CTL

**Figure 8:**
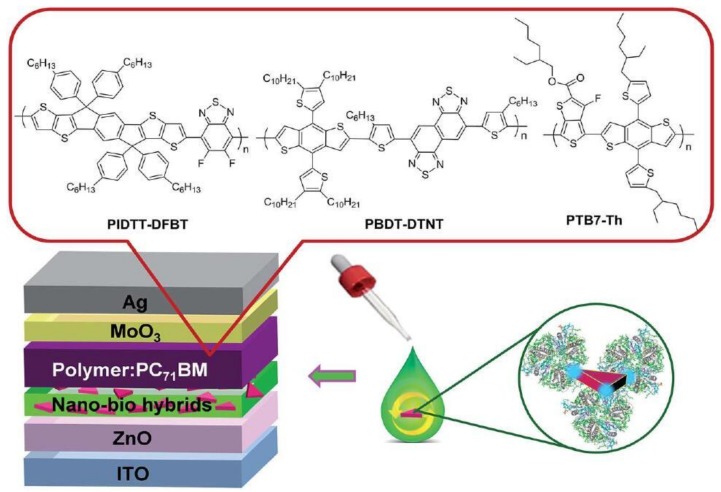
Schematic illustration of the OPV devices incorporating the interlayer of nano-bio hybrids between ZnO and the active layer. The insets show the crystal structure of LHCII, and chemical structures of polymers PIDTT-DFBT, PBDT-DTNT, and PBT7-Th [55]. Reprinted with permission from Ref. [55].

Apart from inside the photoactive layer and CTL, metallic nanostructures can also be placed between two adjacent layers (Figure 8). For instance, the plasmonic nanostructures have been sandwiched between the active layer and CTL. Photons in a broad wavelength band can be harvested by the hybridization of extinction-tailorable Ag nanoprisms with natural photosensitizer protein molecules. The photocurrent is increased by the incorporation of the nano-bio hybrid as an interlayer between the active layer and ETL, which can attributed to LSPR of Ag nanoprisms and the energy transfer from LHCII [55]. Additionally, Au NRs with silica shell thickness of ~3 nm sandwiched between the active layer and HTL improved the *J*_sc_ and PCE of the organic solar cells, primarily due to the strong local electromagnetic field. The attenuation of the LSPR field is mitigated by the SiO_2_ shell, leading to a high local field intensity in the active layer and enhanced absorption of PTB7 [69].

### 4.4. As Electrode

ITO is current widely used as transparent electrode, but it has some disadvantages such as brittleness and indium scarcity [58]. As a promising candidate for the transparent electrode to replace ITO, the metallic nanostructures exhibit several features beneficial to the OPV devices, like increased flexibity and enhanced near infrared (NIR) absorption due to the plasmonic effect.

Ag NRs with excellent optical and electrical properties are promising candidates for future transparent electrodes. However, pure NRs possess low melting point and fail to work at c.a. 100 °C. The Ag NRs networks easily agglomerate at the intersection under thermal stress. In last years, multilayer structures have been developed with the metallic nanostructures sandwiched in between. The multilayer structure may block the migration and thermal-dynamic movement of the metal NRs for aggregation. Therefore, the optical and electrical properties, and thermal stability, may be improved. Furthermore, the two layers on top and beneath the metal layer may help present constructive interference for the electromagnetic field, boosting the plasmonic effect for the device. 

**Figure 9:**
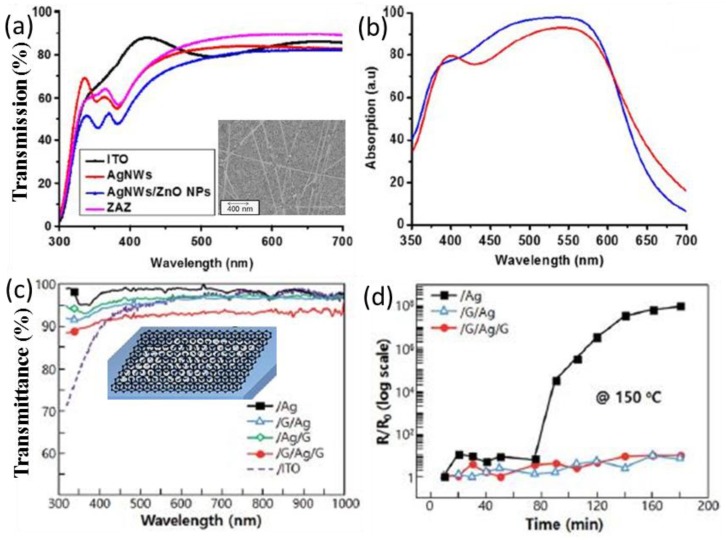
(**a**) Measured transmission spectra of ZAZ, AgNWs, AgNWs/ZnO NPs, and ITO electrodes. The inset is the SEM image of the ZnO-Ag-ZnO electrode; (**b**) calculated absorption inside P3HT:PCBM of organic solar cells integrating ZAZ and ITO electrodes [80]; (**c**) UV-vis transmittance of several Ag NWs electrodes system of /G, /Ag/G, and G/Ag/G films on Si (100)/SiO_2_ substrate, respectively. The inset is schematic illustration of each electrode stack of Graphene/Ag/Graphene; (**d**) change in the relative resistance of electrode materials according to the number of bending cycles [63]. Reprinted with permission from Refs. [63,80].

For instance, Chalh et al. fabricated ZnO NP/Ag NR/ZnO NP (ZAZ) electrodes, which are fully solution processable [80]. Lee et al. transferred graphene both on top and at the bottom of Ag NR networks to protect the nanostructures against thermal degradation [63]. The transmittance of ZAZ surpasses that of ITO at wavelengths larger than 470 nm (see Figure 9). Constructive interference of the incident light in ZAZ produced better transmittance than pure Ag NRs. Whereas the ITO presents low transmittance in the UV region, Ag NR-based flexible electrodes show a uniform transmittance of more than 90% in all wavelength regions. When the top ZnO layer is thin enough (≤11 nm), the electric field in the active layer can be intensified by the plasmonic effect. The mechanical tensile strength and the thermal stability are significantly promoted in the graphene-Ag-graphene (GAG) structure [90,91,92]. Its sheet resistance remains stable when the device is annealed at 170 °C for several hundred hours, while pure Ag NWs degraded only at 150 °C for 1 h [61].

**Figure 10:**
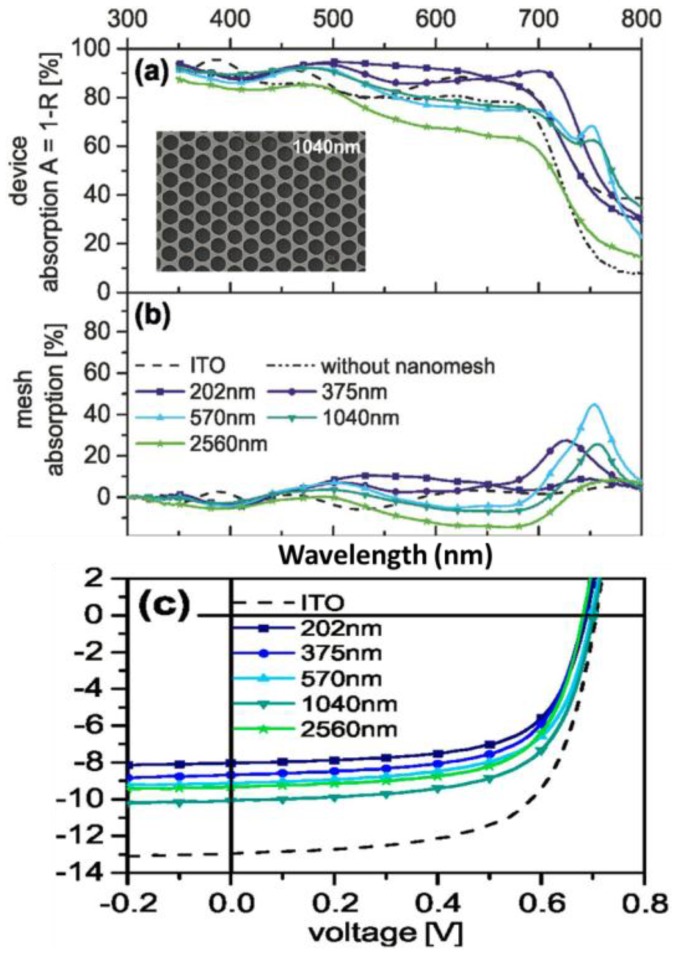
(**a**) Absorption spectra of PTB7:PC_71_BM solar cells with gold nanohole electrodes for different periodicities (solid lines), of the same solar cell without the nanomesh (dash-dotted line), and of the PTB7:PC_71_BM reference device on ITO (dashed line); (**b**) contribution of the nanohole electrode to the device absorption. The nanomesh absorption is obtained by subtracting the absorption (A = 1 − R) of the same solar cell device without a nanomesh electrode (Rblank, dash-dotted line) from the device absorption with nanomesh electrode; (**c**) current-density—Voltage characteristics under illumination of solar cells built on ITO (dashed line) and gold nanohole array transparent conducting electrodes with different periodicities [59]. Reprinted with permission from Ref. [59].

Another alternative substitute for ITO may be metallic nanomeshes, namely, periodic nanohole arrays due to the combining features of fare optical transmittance and the surface plasmonic properties (Figure 10). However, it is still controversial whether the plasmonic nanomesh electrodes can surpass the conventional ITO in OPV efficiencies [59].

## 5. Photophysical Investigations of the Plasmonic Effect

Photophysics is an important link between the structural properties of the diverse layers in organic solar cells and the ultimate device performance. Usually, the photophysical properties are investigated by steady-state and transient optical spectroscopies. The steady-state photophysics, such as the absorption and reflection, are key criteria to judge whether the plasmonic effects take place [51,76]. Normally, the enhancement in the optical absorption and the decrease in reflection are indicators of effective plasmonics due to the far-field scattering and near-field LSPR. It is important to point out at the outset of this part that these optical footprints should be analyzed in association with the electrical properties (e.g., EQE) to unambiguously determine the involvement of plasmonics for boosting the OPV performance.

The steady-state optical spectroscopies have been performed to reveal the fundamental plasmonic mechanisms including various modes such as the scattering, LSPR, SPP, waveguide, and the plasmon-gap mode. In organic solar cells, characteristics like the exciton generation/dissociation and the charge carrier transport/collection, which are crucial to the device performance, can be addressed based on the transient photophysical properties. The charge transfer and transport properties have been studied via monitoring the relaxation kinetics of excitons in excited states with the time-resolved absorption and fluorescence measurements.

Overall, controversial findings have been reported on incorporation effect of the plasmonic metallic nanostructures. Despite the fact that many studies demonstrated the improvement of the photovolatic performance thanks to the introduction of the nanometals, the device performance deterioration shown in some other works should not be ignored. Hence, it is urgently needed to obtain physical insights into the plasmonics from the investigation of the photophysical processes for exciton dissociation into free charge carriers and charge recombination/transport/collection [93,94,95]. In this part, we focus on the photophysical properties in plasmonic photovoltaic structures in correlation with the device configurations and performances.

### 5.1. Steady-State Characterizations of Absorption and Photoluminescence

#### 5.1.1. To Confirm Plasmatic Effects Rendered by the Incorporation of Nanometals

**Figure 11:**
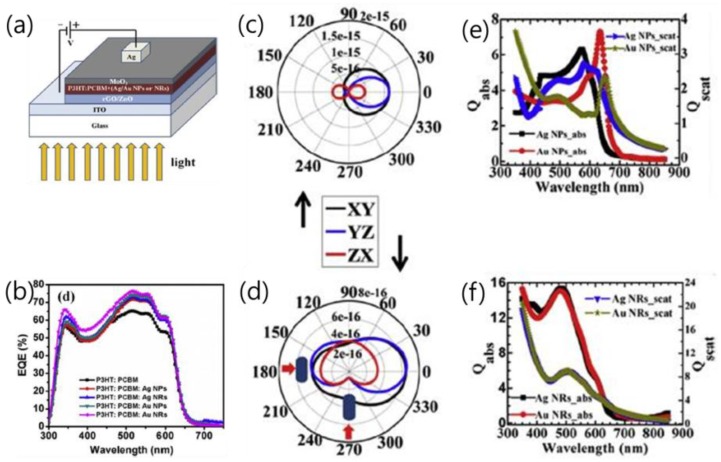
(**a**) Device architecture of ITO/rGO (0.4 wt %) ZnO/P3HT: PCBM: 20 wt % of Ag/Au NPs or NRs; (**b**) EQE spectra of ITO/rGO (0.4 wt %) ZnO/P3HT:PCBM: 20 wt % of Ag/Au NPs or NRs photovoltaic devices (Ag NPs 71%, Ag NRs 73%, 74% for Au NPs and 76% for Au NRs; (**c**,**d**). The angular distribution of scattered radiation in XY, YZ, and XZ planes. The Absorption (Qabs) and scattering (Qscat) cross sections of (**e**) Ag NPs (or) (**f**) NRs at various wavelengths of the incident spectrum [51]. Reprinted with permission from Ref. [51].

When the metallic nanostructures are embedded inside the active layer as shown in Figure 11, the device performance is improved due to the increased absorption, which may arise from the near-field LSPR or/and the far-field scattering. For these plasmonic nanostructures, it is generally believed that the LSPR at the metal/dielectric interfaces is a primary and significant contribution to the enhancement of electric field, and hence the absorption in the light absorbing medium. The near field plasmatic effect can be validated by the FDTD calculation results and can be experimentally supported by the large deviations between the curves of cross sections for the scattering and the total absorption (Figure 11e,f) [51].

Compared with LSPR, the cross section of scattering is not trivial for the Au/Ag NPs/NRs in the wavelength range of 450–650 nm. The nanometals could possess a scattering cross-section much higher than the geometric cross section value. In this case, the absorption is enhanced as a result of the increased optical path length in the active layer caused by the light reemitted in different directions within the device [96]. The angular distribution of the scattered radiations is depicted in Figure 11c,d, in which the Y-axis denotes the direction of forward scattering. The light scattering prefers the forward direction, because the diameters of the Au/Ag NPs are c.a. 40 nm and the NRs are 50 nm in length and 10 nm in width, which is consistent with the criteria described in Ref. [22] for effective forward scattering, i.e., in 30–60 nm range. The NPs and NRs in the active layer function as subwavelength scattering elements and trap the freely propagating light waves. NRs present large absorption cross sections and better *J*_sc_ and EQE than NPs despite the fact that their geometrical sizes are comparable, probably because NRs possess longitudinal and transverse plasmatic modes.

**Figure 12:**
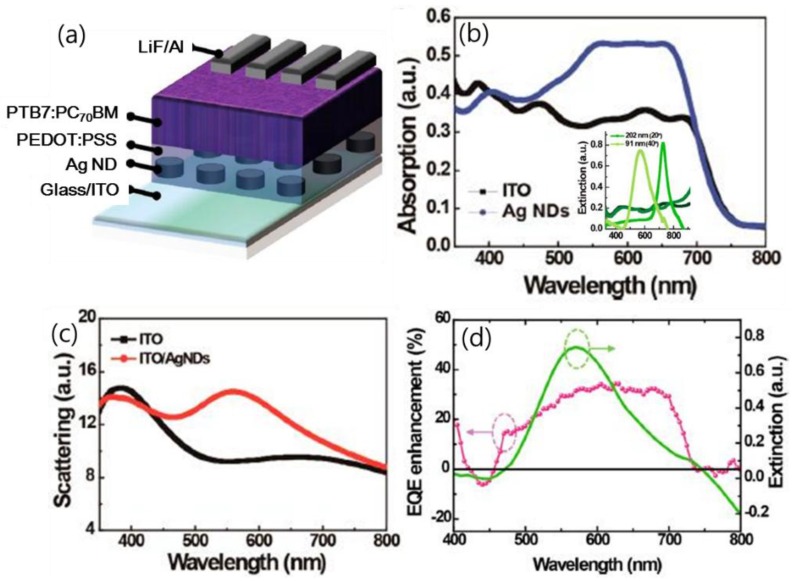
(**a**) Schematic diagram of an OPV device with Ag NDs; (**b**) measured absorption of the PTB7:PC70BM layer coated on different substrates. Absorption of PTB7:PC70BM coated on the glass/ITO substrate is obtained by subtracting the absorption of the glass/ITO substrate from the glass/ITO/PTB7:PC_70_BM substrate (black line). Absorption of PTB7:PC_70_BM coated on the glass/ITO/Ag ND substrate is obtained by subtracting the absorption of the glass/ITO/Ag NDs substrate from the glass/ITO/Ag NDs/PTB7:PC70BM substrate (blue line); (**c**) measured light scattering factor using an integrated sphere system; (**d**) EQE enhancement after embedding Ag NDs in the OPV device and extinction spectra of 91 nm Ag NDs. The wavelength range of EQE enhancement corresponds with the extinction spectrum [76]. Reprinted with permission from Ref. [76].

Upon the incorporation of Ag nanodots (NDs) in the charge transport layers as shown in Figure 12, the optical path and hence the EQE of OPV solar cells are increased. The wavelength bands for the absorption and EQE enhancement match the extinction spectrum of Ag NDs. In view of the location of Ag NDs, which is outside the active layer, the far-field scattering usually takes a remarkable role in the absorption enhancement. It was experimentally validated in the spectra of scattered light measured with the integrated sphere system. Conspicuous scattered light can be observed from 460 to 730 nm. Effective forward scattering rendered by the Ag NDs in the front charge transport layer increases the length of the optical path in the active layer, leading to promoted light trapping and increased *J*_sc_.

One needs to determine whether (and if yes how much) the LSPR mechanism contributes to the absorption enhancement when the nanostructures are away from the active layer. FDTD simulations show that a strong electric field enhancement can be generated in the charge transport layer and can diffuse efficiently into the active layer, confirming the involvement of the LSPR effect in the plasmonic processes.

The weight ratio of the plasmonic contributions from the near-field LSPR and far-field scattering effect may vary with parameters such as the locations and sizes of the nanostructures. In brief, it is generally accepted that LSPR is the main plasmonic mechanism for nanometals hybrided in the active layer, whereas the scattering is the first plasmonic mode to be considered for nanostructures embedded in buffer layers. As calculated in Ref. [70], the EQE enhancement was principally aroused by the far-field scattering, while the LSPR merely contributed to ~0.7% of the enhancement, when Au NRs (30 nm long and 10 nm wide) were dispersed in the rear ETL as a back reflector.

#### 5.1.2. To Elucidate Detailed Plasmonic Mechanisms

In addition to the aforementioned LSPR and scattering effects, there are other plasmonic processes that can be discriminated from the photophysical characterizations.

**Figure 13:**
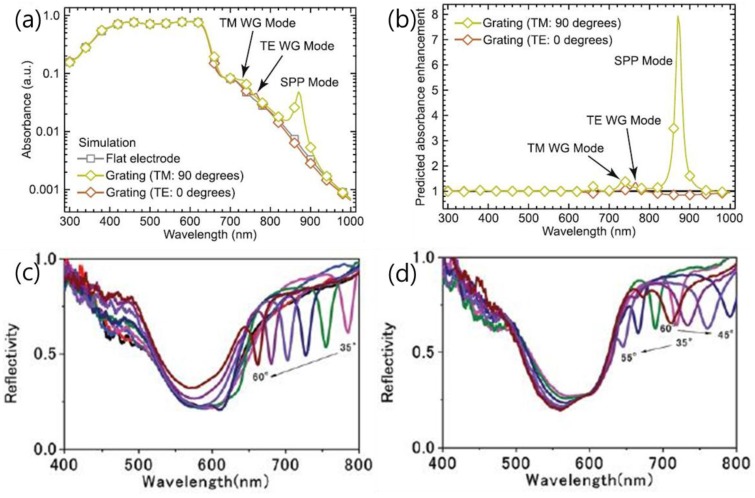
(**a**) Simulated absorbance spectra as a function of the incident light polarization relative to the grating period, for the active layer in a complete device stack incorporating the same Ag grating, with the corresponding absorbance for a device with a flat Ag electrode for comparison [56]; (**b**) the predicted polarization-dependent absorbance enhancement as a result of incorporating the nanostructured Ag grating electrode; (**c**) device EQE enhancement factor (see text for definition) for normal incidence with polarization parallel (TE; left) and perpendicular to the period of the grating (TM; right). Reflectivity measurements of inverted OSCs by illumination with TE (s-pol) (**d**) or TM (p-pol) [81]. Reprinted with permission from Refs. [56,81].

In order to assess the SPP mode, the spectral reflectivity and absorption have been calculated with theoretical simulations based on the structure of P3HT:PCBM thin film on the metallic grating as electrode. In inverted solar cells with configurations of ITO/ZnO/P3HT:PCBM//P3HT:sorbitol/Ag grating/PET [72] and ITO/TiO_2_/P3HT:PCBM/PEDOT:PSS/Au grating [56], both SPP and photonic waveguide modes have been observed. The occurrence of the SPP or waveguide mode is essentially affected by the relative orientation of the incident light polarization to the grating lines. Only with the TM (i.e., in p-polarization, perpendicular with the grating lines) excitation can SPP modes be excited. In contrast, the waveguide mode can be detected under both TM and TE (i.e., in s-polarization, parallel to the grating lines) incident irradiations. Waveguide modes are not as readily observed as SPP, because they may be easily deteriorated by the small thickness of the organic solar cells [56]. The quantity of the waveguide modes increases with the thickness, whereas the SPP mode does not [81] (see Figure 13).

With appropriate selection of the grating pitch, the SPP mode can be tuned to the NIR spectral region. Hence, the wavelength range of the absorption is expanded in the active layer due to the nanostructured electrodes. The light trapping and the optical field in the active layer are effectively intensified by the SPP mode issued by the Ag grating electrode in the OPV device. The spectral position of the simulated SPP mode agrees well with the measured EQE enhancement, demonstrating that the charge transfer states are photoinduced by the plasmatic trapping and can generate free carriers for charge collection when the nanograting is used as electrode [56].

**Figure 14:**
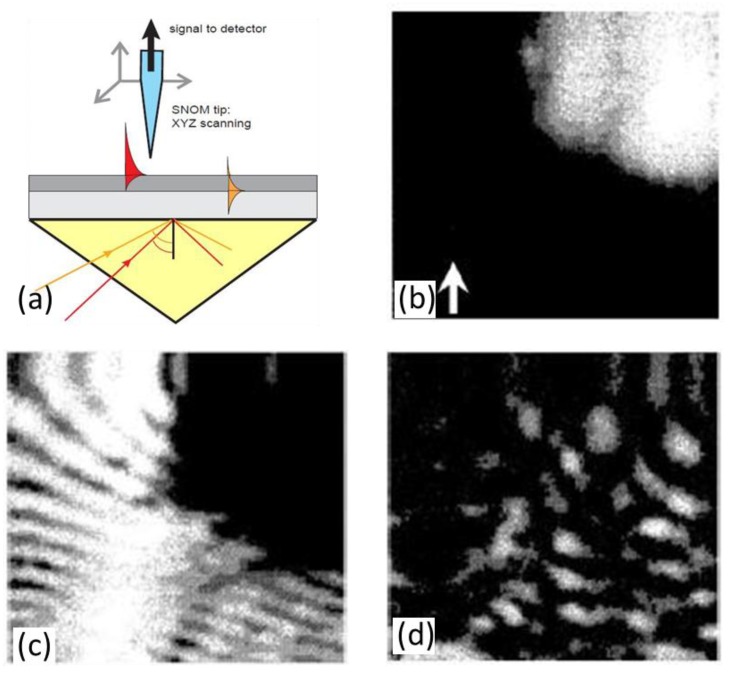
(**a**) Schematic of a SNOM experimental set-up for studying surface plasmon polaritons (SPP) [97]. Topography (**b**), near-field, (**c**) and far-field (**d**) intensity distributions over the smooth gold surface [98]. Reprinted with permission from Ref. [97,98].

Apart from the theoretical simulation, the SPP field can also be monitored in experimental ways. Due to the development of scanning probe techniques, the SPP field can be directly and locally probed on a surface, with a resolution in the nanometer range. The scanning near-field optical microscopy (SNOM) apparatus is illustrated in Figure 14. The incidence angle of the laser can be tuned to excite SPP at desired surfaces, and the evanescent field of SPP is detected by the fiber tip of SNOM. By scanning the fiber tip over some areas, the sample topography and the SPP field distribution can be simultaneously recorded with a SNOM [97,98]. From the topographical image, one can see the smooth interfaces with roughness of about 10 nm except for the bumps with height of ~100 nm. The near-field optical images exhibit typical pronounced interference patterns, which are produced by the interference between the scattered SPP and the SPP on the surface. The signal intensity decreases to 5% if the fiber tip moves by 1 μm away from the surface, indicating the SPP substantially propagates in the lateral direction along the interfaces.

**Figure 15:**
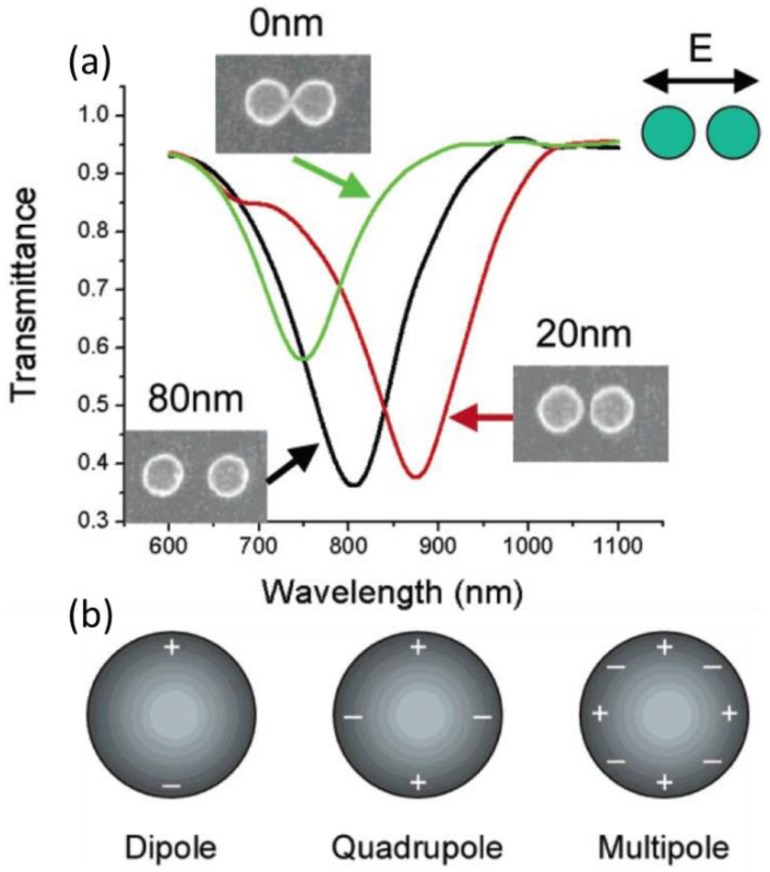
(**a**) Polarized transmission spectra in periodic arrays of pairwise interacting gold nanoparticles. The lattice constant is 800 nm in parallel direction to the pair axis, 400 nm perpendicular, and the dot height is 30 nm; (**b**) pictorial illustration of charge distributions for dipole, quadrupole, and multipole modes for a single spherical particle [99]. Reprinted with permission from Ref. [99].

In organic solar cells, there may exist some additional modes like mirror charge mode or gap mode when periodic nanostructure array are fabricated on or in the back metallic electrode [87]. Transmittance measurements showed the redshift of the spectral position of the transmittance minimum with the decreasing distance between the NP pairs as long as they are not directly linked (see Figure 15). There is an abrupt spectral blueshift once the NP pairs are in contact. This is associated with the competition between the dipolar and quadrupolar contributions for the absorptive cross section in separated Ag NPs. The red shift is as large as ~200 nm when the edge-to-edge distance of the NP pairs is 10 nm. Therefore, taking advantage of the gap mode, one can tune the local electromagnetic field by tailoring the spatial arrangement of the periodic arrays [99]. In devices illustrated in Figure 15c, plasmonic resonances can be generated between the Ag oblate NPs and the active layer. In addition to LSPR, plasmonic gap mode is formed in the gaps between the adjacent NPs and between NPs and anode [77]. The efficient hybridization of the LSPR and the gap modes can provide multiplicative enhancement of absorption over a broad band that tolerates a wide selection of grating period, e.g., from 190 to 400 nm [100].

### 5.2. Transient Absorption and Photoluminescence Spectroscopies

**Figure 16:**
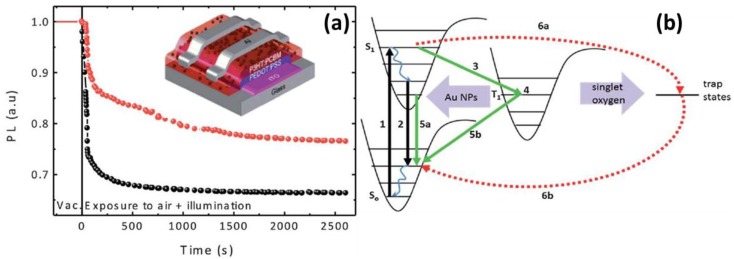
(**a**) PL decay measurements: comparison of the pristine (black) and NP-doped blend (red). The inset is the schematic device architecture of the solar cell. (**b**) Schematic of the photo-oxidation process in the polymer: Au NPs active layer. Energy from the polymer triplet excitons excites singlet oxygen, which reacts with the polymer chains to form exciton trap states. The Au NPs embedded into the blend act as quenchers of the triplet excitons, and in this way the photooxidation process can be impeded. (1) Absorption; (2) luminescence; (3) system intercrossing; (4) triplet state; (5a, 5b) triplet quenching; (6a, 6b) exciton recombination via trap states. This process is limited in the presence of a triplet quencher [101]. Reprinted with permission from Ref. [101].

Paci et al. incorporated Au NPs into the active layer P3HT:PCBM of the organic solar cells, achieving an enhancement of PCE by 42% and improvement in stability. From the PL decay profiles, one can see the fluorescence decay of the polymer is retarded by the addition of Au NPs (Figure 16). The authors attributed the increased fluorescence lifetime to the interaction between the comparatively long-lived triplet excitons of P3HT as the donor and the Au NPs as the acceptor. Such interactions can strongly quench the triplet state and ameliorate the photo-oxidation of the organic materials. The mitigation mechanism for the device degradation as unveiled by the photophysical characterization is supported by the time resolved energy dispersive X-ray reflectometry (EDXR) study [101].

**Figure 17:**
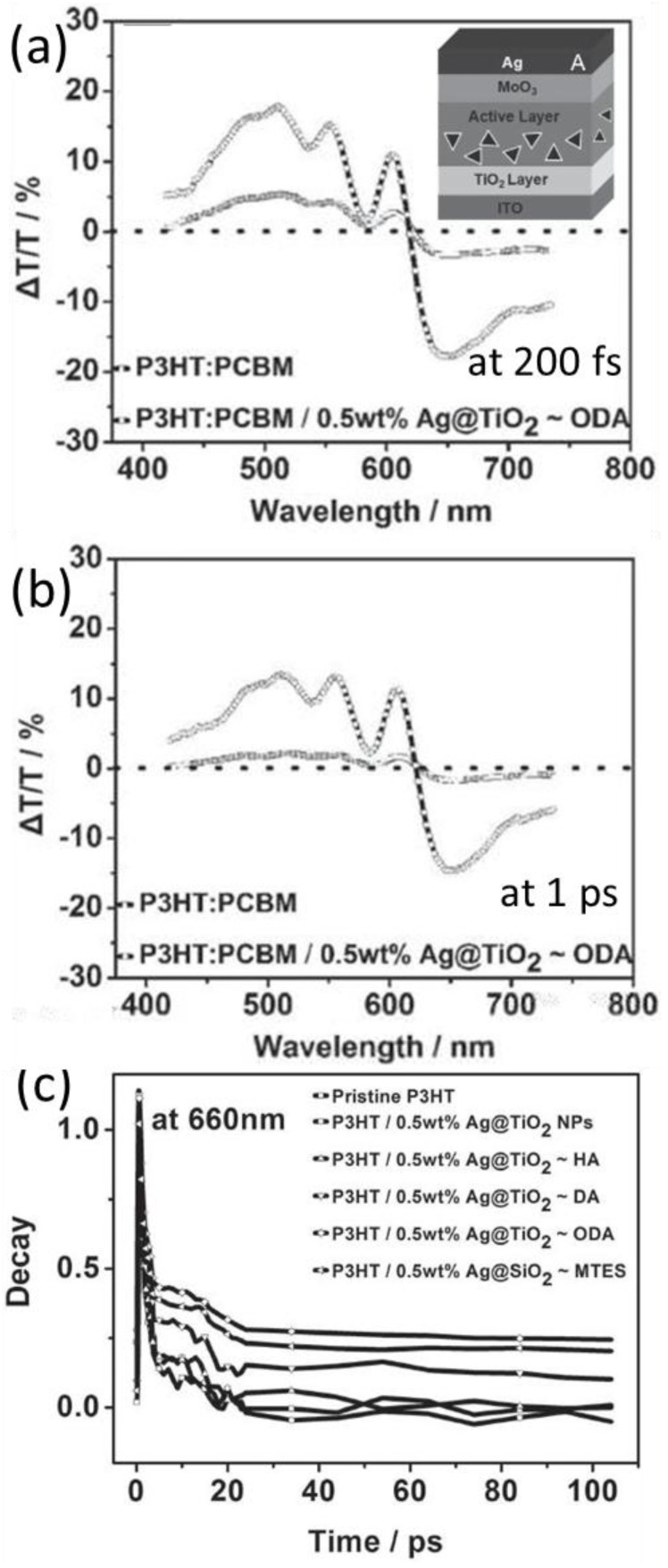
Relative differential transmissions of P3HT:PCBM films with delay times of (**a**) 200 fs and (**b**) 1 ps after excitation with 400 nm laser beam. (**c**) Transient decays of P3HT film, P3HT blended with Ag NPs, and P3HT blended with Ag@oxides nanoprisms. PIA decay monitored at 660 nm [93]. Reprinted with permission from Ref. [93].

Since the metallic NPs embedded in the active layer may increase the trapping of polarons and recombination of excitons, limiting the amount of free charge carriers, ultrathin layer of oxide can be added to encapsulate the NPs. The femtosecond transient absorption results of P3HT:PCBM and their blends with Ag@TiO_2_ at 200 fs and 1 ps delay are displayed in Figure 17a,b. The films exhibited three vibronic peaks in the wavelength range from 520 to 610 nm, which arise from ground-state bleaching (GSB). The negative peak centered at 660 nm originates from the photoinduced absorption due to polaron pairs according to Korovyanko et al. [102]. The increase of this peak for the 200 fs and 1 ps delay suggests that polaron pair generation is more efficient in the hybrid system with the capped Ag NPs than in P3HT:PCBM film.

Consistently with the above Au NPs case, the relaxation time of the photoinduced polarons is also prolonged when Ag@oxides NPs are hybridized in the active layer. This is caused by the enhanced absorption due to LSPR, which is beneficial for the generations of charge carriers. Hence, the P3HT:PCBM-based organic solar cells with Ag@TiO_2_-ODA nanoprisms in the active layer achieved a dramatically improved PCE of 4.03% with enhanced absorption over a broad spectral range of 400–620 nm [93].

**Figure 18:**
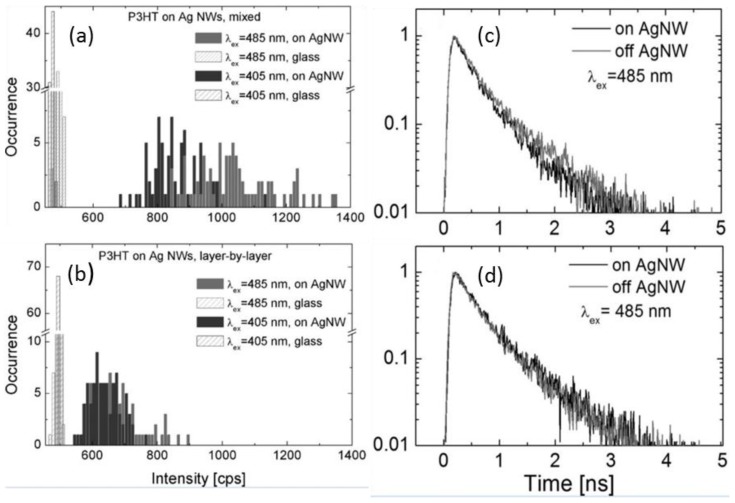
(**a**) Normalized fluorescence transients for samples where AgNWs were (**a**) mixed with PEDOT:PSS and (**b**) covered with PEDOT:PSS prior to deposition of the P3HT layer. Red curves correspond to data measured off the nanowires, while black curves represent data obtained with a laser spot placed at the nanowire. The excitation energy of 485 nm was used; (**c**) fluorescence intensities of P3HT on and off AgNW for the sample where AgNWs were mixed with PEDOT:PSS prior deposition of P3HT; (**d**) fluorescence intensities of P3HT on and off AgNW for the sample where PEDOT:PSS was deposited on AgNWs prior deposition of P3HT. In both cases, the results obtained for lasers excitations of 405 nm (blue bars) and 485 nm (red bars) are displayed [94]. Reprinted with permission from Ref. [94].

PEDOT:PSS is a popular hole transport layer in OPV devices. Metallic nanostructures doped in the HTL or inserted at the HTL-anode interface may contribute plasmonic improvement for the photovoltaic devices. The photophysics of P3HT has been explored in two configurations where Ag NRs were mixed with PEDOT:PSS or covered with PEDOT:PSS prior to the deposition of the P3HT layer. As shown in Figure 18, the P3HT fluorescence intensity is larger on Ag NRs than that off the NRs. The fluorescence emission is intensified due to the enhanced absorption of P3HT aided by the plasmonic interaction between Ag NRs and P3HT. The PL intensity changes with the varying distance between them. Compared with the case where Ag NRs are inside the HTL, the P3HT-Ag NRs distance is more homogeneous but larger when Ag NRs were covered under the HTL. Therefore, the PL intensity and thus the absorption are comparatively more homogeneous but smaller when the nanometals are between the HTL and anode. Hence, there is a compromise between the homogeneity and strength of the metal-polymer plasmonic interactions. This can serve as experimental evidence to support the theoretical simulation conclusion that the LSPR may diffuse into the active layer for absorption improvement even when the metallic nanostructures are placed outside the active layer. If the enhancement of the optical absorption had only been caused by the far-field scattering effect of the Ag NRs, the amplitude of fluorescence intensity for the two geometries would have been quite similar [94].

The transient fluorescence decay profiles are displayed in Figure 18c,d. There is no clearly visible variation to the fluorescence decay times for P3HT on and off the Ag NRs, suggesting there is no direct charge transfer between the polymer and the Ag NRs. It is different from the case when the nanometals are blended in the P3HT:PCBM active layer for which the charge transfer between the polymer and the metal nanostructures gives rise to distinct change of PL decays.

**Figure 19:**
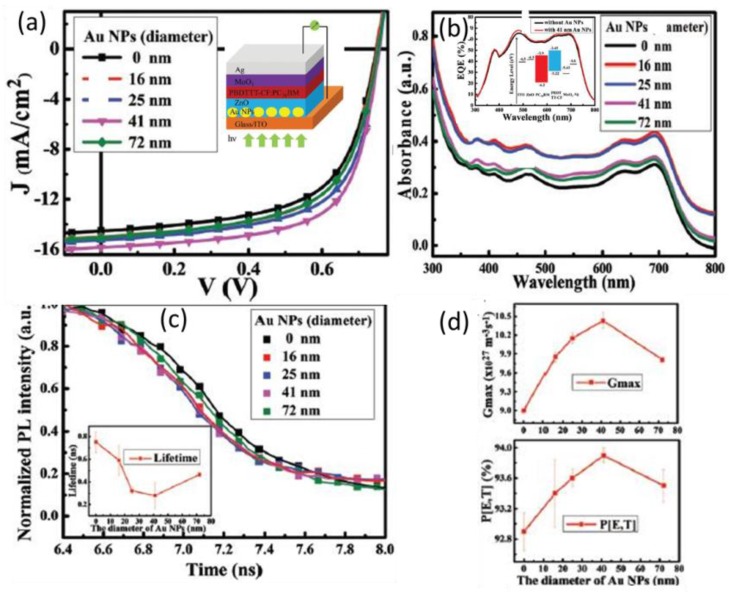
(**a**) J-V curves of the devices with various Au particle sizes. The inset is the schematic of the device structure. (**b**) Absorbance spectra of the structure of ITO/Au NPs:ZnO/PBDTTT-CF:PC70BM with various Au particle sizes. The inset is EQE spectra of the device without Au NPs and the device with 41 nm Au NPs. (**c**) Transient PL decays of the structures of ITO/Au NPs:ZnO/PBDTTT-CF:PC_71_BM with different sizes of Au NPs. The inset gives the dependence of the lifetime on the diameters of Au NPs. (**d**) The dependence of the maximum exciton generation rate Gmax and exciton dissociation probability P(E, T) on the diameters of Au NPs [73]. Reprinted with permission from Ref. [73].

However, it is possible to induce charge/energy transfer between the donor in the active layer and the nanometals in CTL. Chi et al. reported the photophysical process for metallic NPs in ETL as shown in Figure 19. The size of the NPs was manipulated to vary from 16 to 72 nm. The absorption of the plasmonic structures is enhanced due to the scattering and LSPR effects. Although the optical absorption of the whole solar cell reached its maximum with Au NPs of 16 and 25 nm, the device PCE had the highest value, i.e., 7.86%, when Au NPs with diameters of 41 nm were doped in the ZnO ETL. Apart from the absorption, the efficiency of exciton dissociation into free charge carriers is another vital influencing factor to the device performance, which can be explored by time-resolved PL decay characterizations. The PL from the plasmonic samples decays faster than the reference structure, indicating Au NPs improve the exciton dissociation into free holes and electrons. The fluorescence lifetimes have been attained by a bi-exponential fitting, whose dependence on the particle size is presented as the inset of Figure 19c. Therefore, Au NPs with appropriate sizes are able to increase the absorption and exciton separation, giving rise to improved *J*_sc_ and PCE. Too small NPs cannot convert the absorbed photons efficiently into free charge carriers. Too large NPs may induce a high roughness of the layer morphology, deteriorating the OPV devices.

**Figure 20:**
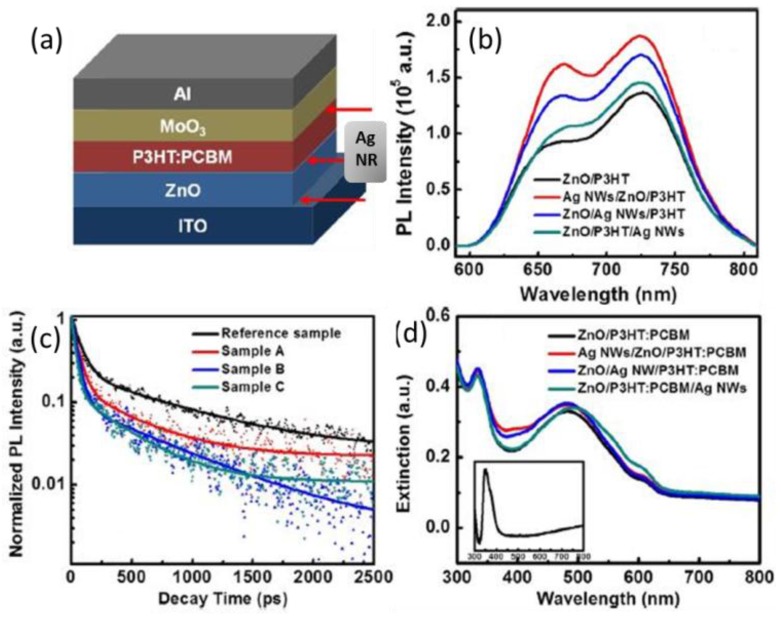
(**a**) Device architecture of the reference inverted PSCs (without Ag NWs) or the plasmonic inverted PSCs (with Ag NRs); (**b**) PL spectra of the reference and plasmonic samples recorded using excitation source wavelengths of 500 nm; (**c**) PL decay profiles for the P3HT:PCBM blends in the reference and plasmonic samples. Excitation source: 500 nm pulsed laser; (**d**) extinction spectra of the reference and plasmonic samples. Inset shows extinction spectrum of Ag NWs [79]. Reprinted with permission from Ref. [79].

In addition, metallic nanostructures can be located between neighboring layers, and the photophysical results can well explain the device performance enhancement. Yang et al. analyzed the steady-state and time-resolved PL curves of P3HT for samples with Ag NRs at ITO/ETL, ETL/active layer, and active layer/HTL interfaces, respectively, as exhibited in Figure 20. From the steady-state PL spectra in Figure 20b, the Huang-Rhys factor (*S* = *I*_0–1_/*I*_0–0_) is obtained as an indicator of the conjugation length of polymers. The S value is the smallest when Ag NRs are between ITO and ETL. The optical absorption is amplified to the greatest extent with the same plasmonic architecture. The transient PL profiles presented faster decays for plasmonic samples compared with the reference one (see Figure 20c), demonstrating strong coupling of the excitons and plasmonic field. The photophysical characterizations manifest that the exciton dissociation efficiency, the electron delocalization, and the absorption are improved by the Ag NRs, in particular when they are at ITO/ETL interface. With this optimized plasmonic structure, *J*_sc_ is increased and *V*_oc_ and FF remain almost unchanged. The PCE of P3HT:PCBM solar cells is increased from 3.10% for the control device to 4.05% in the plasmonic device with Ag NRs inserted at the ETL/cathode interface [79].

**Figure 21:**
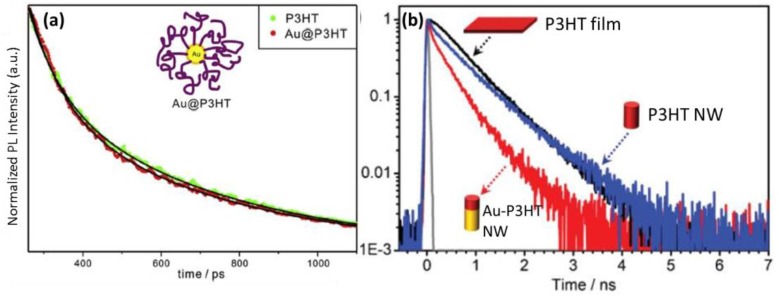
(**a**) Picosecond time-resolved fluorescence kinetic profiles of P3HT and Au@P3HT in chloroform. Samples were excited at 355 nm and monitored at 580 nm, and solid lines are the best-fitted curves to extract kinetic constants. The inset is the schematic structure of Au@P3HT nanocomposite [103]. (**b**) PL lifetime decays of a P3HT thin film (black), a single P3HT nanowire (blue), and a gold-P3HT nanowire (red) along with the instrument response function (IRF) of the PL lifetime system (grey) [95]. Reprinted with permission from ref [97]. Reprinted with permission from Refs. [95,103].

Additionally, organic-inorganic hybrid nanostructures comprised of polymer semiconductors and plasmonic metal have been newly developed for future photocatalytic and photovoltaic applications [95,103]. The nanostructures hold the polymer and metal in close proximity, facilitating the charge/energy transfer and the exciton dissociations that are beneficial to charge collection in OPV devices. For instance, Au@P3HT nanocomposites and Au-P3HT NRs have been fabricated, and the time-resolved PL profiles are illustrated in Figure 21. The mean decay time of Au@P3HT composite is shorter than that of pristine P3HT. Analogously, Au-P3HT NRs possess notably decreased fluorescence lifetimes compared to neat P3HT NRs [85]. The increased fluorescence decay rate of semiconductor polymers in the presence of plasmonic metals is associated with the energy transfer from the excited state of P3HT to the SPR state of gold NPs [103].

**Figure 22:**
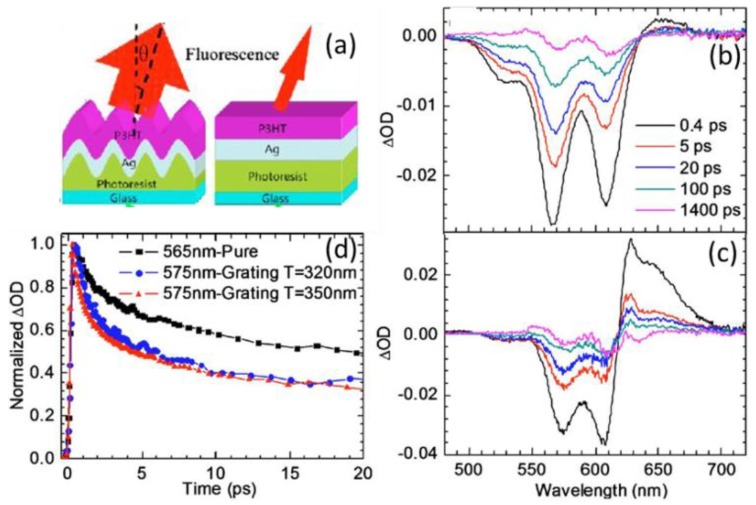
(**a**) Schematic of the fabricated samples with the structure of glass. TA spectra recorded at 0.4, 5, 20, 100, and 1400 ps, respectively, of P3HT on a (**b**) flat Ag film and (**c**) Ag grating. (**d**) TA dynamics at one peak of the photobleaching spectrums [104]. Reprinted with permission from Ref. [104].

As illustrated in Figure 22, the transient absorption spectra from P3HT on a flat Ag film and on Ag gratings were compared. Whereas the GSB signals distribute from 500 to 620 nm, the positive peak around 660 nm is attributed to the polaron pair absorption. From the comparison of the peak at 660 nm, one can see that much more polaron pairs are generated via E-E annihilation on samples with Ag gratings, which may be correlated to the enhanced density of the singlet excitons. Owing to the involvement of periodic Ag gratings, the absorption in P3HT is enhanced, and hence more singlet excitons are generated. In Figure 22d, the normalized bleaching recovery of P3HT decays faster when it is on Ag grating, suggesting that the E-E annihilation is enhanced due to the improved absorption in P3HT [104].

## 6. Conclusions and Outlook

In summary, we reviewed in this work the recent advances of plasmonic OPVs and relevant photophysical investigations, which reveal the underlying physics involved in the photovoltaic processes. Important information can be provided by the photophysics to unveil the plasmonic mechanisms and the charge transfer dynamics. It is worth noting that the electrical parameters should be monitored in parallel to the photophysics to make sure the plasmonic absorption enhancement in deed contributes to the charge generation and device performance improvement.

The increased optical absorption, extended wavelength range, and efficient exciton dissociation can be achieved by the plasmonic effect when metallic nanostructures are embedded within the OPV devices. The plasmonic effect is strongly dependent on the distance of the nanostructures to the active layer. The effects are distinct for nanostructures within or between diverse layers in OPV devices. In thick organic solar cells that are swiftly developing for the future large-scale production of printable photovoltaic devices, the position of the nanostructures can be finely manipulated in certain sublayers of the light absorbing medium.

The detailed plasmonic mechanisms are determined by the device configuration and the location of nanometals in organic solar cells. In addition to the aforementioned plasmonic modes like the scattering, LSPR, SPP, waveguide, plasmonic-cavity, and plasmonic-gap mode, the couplings of the plasmonics with upconversion, or fluorescence resonance energy transfer (FRET), are also potentially useful routes to boost OPV performances [60,105].

A broadband absorption may be obtained in various ways such as the incorporation of multiple categories of nanometals or hybrid metallic nanostructures with non-metal functional molecules. The utilization of novel asymmetric nanostructures has appeared to be a simple and efficient approach to get a broadband absorber. These nanostructures are usually placed in CTL or between the active layer and CTL. In addition, periodic 2D nanoarrays or gratings in CTL or electrodes may also extend the absorption to the NIR range.

Increasing attention should be paid to the application of 2D materials in OPV devices. Graphene and its oxide (GO), and transition metal dichalcogenides (TMDCs), are among the common 2D materials that can be considered in the photovoltaic engineering. Nanosheets of TMDC materials like MoS_2_, WSe_2_, TiTe_2_, and boron nitride (BN) possess a laminar structure, and their electrical properties can be tuned from semiconductors to metals by controlling the number of layers in the sheet.

The plasmonics from non-metal materials have emerged as a subject of increased attention in recent years to overcome the drawbacks of the coinage metals such as the optical loss due to the interband transitions and the high cost due to the scarce supply of Au, Ag, and Cu. For instance, the SPR frequency of graphene is in the infrared and THz range, and the excitation of graphene plasmons by visible light has been reported. Nevertheless, the graphene plasmonics have scarcely been employed in organic solar cells. Although rGO was incorporated in the CTL of the solar cells, the plasmonic effect was not discussed in correlation with rGO. Viable approaches still need to be developed to discriminate unambiguously between the plasmonics from metals and non-metal materials.

## Figures and Tables

**Table 1:** Performance of OPV solar cells with various nanostructures at different locations.

System	Configuration	Mechanism	Short-Circuit Current *J*_sc_	Open-Circuit Voltage (*V*_oc_)	Fill Factor (FF)	PCE	Enhancement
P3HT: PCBM: Ag NPs [68]	control In active layer	LSPR	8.67 10.41	0.60 0.60	61.4 62.8	3.19 3.92	~23% in PCE
P3HT:PCBM:Ag NPs/NRs in active layer [51]	control Ag NPs Ag NRs Au NPs Au NRs	LSPR	9.49 10.60 10.99 11.17 12.21	0.63 0.63 0.63 0.63 0.63	63.47 63.61 63.64 63.70 63.75	3.77 4.21 4.37 4.44 4.85	~28% in PCE
PCDTBT:PC_71_BM: WS_2_-Au [52]	control In active layer	LSPR	10.6 12.3	0.89 0.89	60.2 58.4	5.6 6.3	~13% in PCE
PBDT-TS1:PC_71_BM Au nanostars in active and PEDOT [53]	control In active and HTL	asymmetric modes	18.37 19.24	0.81 0.81	67.00 67.70	9.97 10.50	~5% in PCE
PEDOT/Au NR@ SiO_2_/PTB7:PC_71_BM [69]	control between CTL and active layer	Scattering, LSPR	16.5 21.2	0.74 0.74	0.60 0.60	7.52 9.55	~28% in PCE
PCDTBT:PC_71_BM/Au NRs in TiOx PTB7:PC_71_BM/Au NRs in TiOx [70]	control Au NRs in back ETL control Au NRs in back ETL	Backward scattering Backward scattering	10.87 12.03 16.27 17.17	0.89 0.89 0.76 0.76	61.7 62.9 60.1 61.4	5.96 6.75 7.43 8.01	~13% in PCE ~8% in PCE
PTB7:PC_71_BM/ ZnO@CNT-Au (ETL) [71]	control ZnO@CNT-Au as ETL	Forward scattering	16.18 16.81	0.717 0.721	60.7 64.7	7.0 7.9	~13% in PCE
PCDTBT:PC_71_BM/ZnO (ETL) [72]	control Au arrows in ETL	Forward scattering, LSPR	14.70 17.40	0.85 0.85	49.1 52.9	6.14 7.82	~27% in PCE
PBDTTT-CF:PC_71_BM/ZnO (ETL) [73]	control Au NPs in ETL	Forward scattering, LSPR	14.49 15.81	0.75 0.75	61.7 66.2	6.67 7.86	~18% in PCE
P3HT:ICBA/WO_3_ (HTL) [62,74]	control Cu NPs in rear HTL	Backward scattering	8.71 11.79	0.87 0.87	61.4 62.2	4.65 6.38	~37% in PCE
P3HT:ICBA/ WO_3_ (HTL) [54]	control Ag-Au bimetallic NPs in rear HTL	Backward scattering	7.91 11.01	0.87 0.87	64.6 67.6	4.57 6.55	~43% in PCE
P3HT:PC_61_BM/Ag grating in PEDOT [75]	control tapered Ag grating in HTL	LSPR	12.1	—	—	—	~47% in Jsc
PTB7:PCBM/PEDOT/Ag nanodot array/ITO [76]	control between HTL and anode	LSPR, forward scattering	17.43 23.26	0.75 0.73	0.60 0.61	7.70 10.72	~39% in PCE
Ag networks /ZnO/PCDTBT:PCBM/ MoO_3_/Ag oblate NPs/anode [77]	control Ag oblate NPs array between HTL and anode	hybridization of LSPR and plasmonic gap mode	9.32 11.37	0.882 0.881	63.5 60.0	5.22 6.01	~15% in PCE
PTB7:PC_71_BM/ZnO/Au NPs/ITO [37]	control Au NPs between ETL and cathode	MDM absorber	15.53 15.69	0.72 0.72	60.70 63.99	6.75 7.27	
PTB7:PC_71_BM/nano-bio hybrid/ZnO/ITO [55]	control Ag prisms-LHCII between the active layer and ETL	LSPR	16.01 17.99	0.79 0.80	0.72 0.73	9.03 10.57	~17% in PCE
PBDTTT-C:PC_60_BM/Au NPs/PEDOT/ITO [78]	control Au NPs ~15 nm between the active and HTL	LSPR	10.62 11.74	0.73 0.73	59.9 63.4	4.78 5.52	~15% in PCE
ITO/ZnO/P3HT:PC_61_BM/MoO_3_/Al [79]	reference Ag NWs between cathode and ETL Ag NWs between ETL and active layer	LSPR	8.13 9.87 8.92	0.60 0.61 0.60	0.62 0.63 0.62	3.10 4.05 3.31	~31% in PCE
P3HT:PC_61_BM/ITO (or ZAZ) [80]	control ZnO/Ag NWs/ZnO as transparent electrode	Higher tranmission above 450 nm	9.75 11.6	0.58 0.54	57 57	3.16 3.53	~12% in PCE
P3HT:PC_61_BM/PEDOT/Au (flat or grating) [81]	control Au grating as rear electrode	SPP, photonic waveguide mod	6.13 6.83	0.63 0.63	0.59 0.62	3.03 3.53	~16% in PCE
ITO/ZnO/P3HT:PCBM/Ag grating [56]	control Ag grating as rear electrode	SPP, photonic waveguide mode	factor of ca. 5 enhancement of the EQE				
P3HT:PCBM/Al grating [57]	control Multidiffractive Al grating under light absorbing medium	coupling of SPP modes	absorption in NIR range				
